# Screening for adult ADHD using brief rating tools: What can we conclude from a positive screen? Some caveats

**DOI:** 10.1016/j.comppsych.2021.152224

**Published:** 2021-04

**Authors:** Samuel R. Chamberlain, Samuele Cortese, Jon E. Grant

**Affiliations:** a Department of Psychiatry, Faculty of Medicine, University of Southampton, UK; b Southern Health NHS Foundation Trust, Southampton, UK; c Department of Psychiatry, University of Cambridge, UK; d Center for Innovation in Mental Health, School of Psychology, Faculty of Environmental and Life Sciences, University of Southampton, UK; e Clinical and Experimental Sciences (CNS and Psychiatry), Faculty of Medicine, University of Southampton, UK; f Solent NHS Trust, Southampton, UK; g Division of Psychiatry and Applied Psychology, School of Medicine, University of Nottingham, Nottingham, UK.; h Department of Psychiatry, University of Chicago, USA; i Hassenfeld Children's Hospital at NYU Langone, New York, NY, USA

**Keywords:** Impulsivity, Screen, Screening, ADHD, Hyperkinetic, Inattentive, Impulsive, Sensitivity, Specificity, ASRS

## Abstract

Adult Attention-Deficit/Hyperactivity Disorder (ADHD) is prevalent but often overlooked and undertreated. Left untreated, it is linked to increased risk of untoward outcomes including unemployment, relationship breakups, substance use, driving accidents and other mental health conditions. Several brief screening tools have been developed for adult ADHD. The most frequently used is the World Health Organization's Adult ADHD Self-Report Scale (ASRS V1.1). Here, we show in two independent population samples (UK: *N* = 642, USA: *N* = 579) that the tool resulted in considerable overestimation of ADHD, indicating probable ADHD in 26.0% and 17.3% of participants, as compared to expected prevalence of 2.5%. The estimated positive predictive value was only ~11.5%. Both samples had normal levels of trait impulsivity as assessed using the Barratt Impulsiveness Scale. The data indicate that using the ASRS in general population samples will result in 7–10 times over-identification of ADHD. We use these results to highlight how such tools should most appropriately be used. When being used to determine possible cases (such as for onward referral to an ADHD specialist) they should be complemented by clinical assessment – we give examples of how non-specialists might determine this. When measuring ADHD symptoms dimensionally, researchers should be mindful that the ASRS captures impulsive symptoms other than those due to ADHD. Lastly, we note the need to screen for impulse control disorders (e.g., gambling disorder) when using such tools to measure ADHD, be it for onward referral, or for dimensional research studies.

## Highlights

• ADHD commonly persists into adulthood.
• We deployed the most widely used screening tool for adult ADHD in two normative samples
• The screening tool considerably over-identified probable ADHD, with low positive predictive value.
• Key advice is provided about the appropriate use of such tools, to maximise benefits and minimise harms.

## 1 Short communication

Attention-Deficit/Hyperactivity Disorder (ADHD) is the most common neuropsychiatric disorder of childhood, with impairing symptoms persisting into adulthood in approximately 70% of cases [1]. A meta-analysis of international data estimated the prevalence of ADHD in adults to be around 2.5% [2]. Adequate detection of ADHD is important since when left untreated, it is associated with major untoward functional consequences including driving accidents, worse scholastic outcomes, unemployment, criminality, and risk of developing other mental health conditions (including substance use problems) [3,4]. First-line treatments for adult ADHD (i.e., stimulants) have a strong evidence base, with medium-large effect size improvements versus placebo in the short to medium term [5]. Such evidence-based treatments can mitigate untoward outcomes for sufferers [3,4]. At the same time, avoiding misdiagnosis / over diagnosis of ADHD is vital: stimulants have diversion and abuse liability in some cases. Also, there is a need to avoid excessive specialist referrals for ADHD assessments for those unlikely to actually have the condition. Inappropriate referrals can result in distress for the person referred (e.g. long waiting lists, lengthy assessments, and then finding out they do not have ADHD), and also result in higher healthcare costs.

For clinical purposes, ADHD should be diagnosed by a mental health specialist, using structured clinical instruments, taking into account self-report as well as confirmatory collateral reports of childhood and adult symptoms. As well as ensuring interventions are targeted to those most in need, this also reduces the risks of inappropriate prescribing and diversion of stimulants for non-medical uses. However, given that the prevalence of adult ADHD is 2.5% [2], and that healthcare resources are finite, methods are needed by which potential ADHD cases can be conveniently screened prior to extensive clinical interviews. Additionally, methods of identifying probable ADHD using self-report tools are needed so that the disorder can be studied reliably in at-risk populations and the general population. Detailed interviews are typically not feasible for such research studies. Therefore, it is important to consider the specific use of screening instruments and what they can, and cannot, tell us.

The World Health Organization (WHO) Adult ADHD Self-Report Scale v1.1 (ASRS v1.1) Part A is the most widely used screening tool for ADHD cases in adults [6]. The tool is listed in national and international ADHD guidelines e.g. [7]. The instrument comprises six questions covering inattentive and hyperactive-impulsive symptoms, and a threshold of four or more is used for probable ADHD [6]. The score is determined based on the number of questions meeting specific criteria: endorsing sometimes/often/very often for questions 1–3; and endorsing often/very often for questions 4–6. Initial validation indicated that this screener had sensitivity of 68.7% and specificity of 99.5% for detection of ADHD, and the instrument was deemed suitable for use in the general population [6].

We included ASRS v1.1 Part A in two non-treatment seeking normative cohorts of young adults: one in the UK (*N* = 642), and one in the USA (*n* = 579). This was one of several self-report scales included for studies exploring latent phenotypes of impulsivity and compulsivity. For both cohorts, those receiving treatments for any mental health conditions were excluded at entry. Using the standard threshold, the rates of probable ADHD in these two cohorts based on the ASRS v1.1 Part A were 26.0% (UK) and 17.3% (USA) respectively. Given that the expected rate of ADHD was 2.5%, this indicates that 86–90% of people identified as having probable ADHD in these two normative cohorts were unlikely to have ADHD. The estimated positive predictive value (assuming the actual proportion of true cases was in line with expectations) was only ~11.5%.

The two cohorts had normative levels of trait impulsivity as determined by the Barratt Impulsivity Scale (average scores within 0.5SDs of previously published norms in healthy controls). As such, and given that the UK dataset was follow-up from an epidemiologically representative cohort recruited using stratified sampling, it seems unlikely that actual levels of ADHD in the two datasets would deviate meaningfully from population expectations. However, a limitation is that we did not conduct gold-standard clinical interviews for ADHD.

These findings suggest that a positive screen will often not reflect ADHD, and the tool did not show high specificity. This is extremely important for all users of the screener to be aware of. If this tool was used as the sole means of identifying people who should have a specialist ADHD assessment, healthcare resources would be overstretched and many individuals would be subjected to referral, waiting times, and in-depth interviews when it is unlikely they have the diagnosis. Therefore, we recommend that the tool only be used as a screener for categorical ADHD, in people for whom there is high clinical suspicion of ADHD. Examples of ensuring high clinical suspicion that could be used by non-ADHD experts would include, for example checking:1) that there is collateral support for symptoms (e.g., from the patient's partner or family member), 2) symptoms were evident in childhood, 3) symptoms occur in two or more functional domains (e.g., at work and at home), and 4) for other obvious types of disorder that could better account for the problems reported.

Why might people screen positive on ADHD rating tools when they do not have ADHD? First, of course all rating tools have limitations (including psychometrically) relative to clinical interview by a specialist. Even gold-standard diagnoses are not always accurate, since there is an element of subjectivity, and mental health disorders are likely not ‘one thing’ but reflect different biological processes. Second, we suspect many cases of apparent positive self-reported ADHD are due to other conditions such as impulse control disorders and obsessive-compulsive disorders. These are very rarely screened for, but validated convenient screening tools do exist. As such, an accurate differential diagnosis is pivotal. Third, ADHD exists along a continuum and it can be challenging to know where to draw the line: for a diagnosis there must be impairment in at least two distinct life domains (e.g. in the workplace, and in the home) but screening tools do not typically capture that.

Overall, we would suggest that the ASRS v1.1 Part A remains a good choice of the available brief screening tools for ADHD suitable for general population use, but users need to be aware a positive screening outcome will often (indeed if used as the sole means, in the majority of cases) not reflect ADHD. We highlight the cardinal importance of considering other diagnoses, and for rigorously screening for the same if an ADHD diagnosis is being considered. The ASRS v1.1 Part A is also useful to generate a ‘total score’ to provide a dimensional measure of ADHD symptoms to explore associations with such symptoms, rather than as a binary thresholding tool. However, again it is important for researchers to be mindful it likely reflects not only ADHD, but also a multitude of other impulse symptoms for other disorders. Overall, from a research point of view, in view of the very high apparent rates of ADHD detected in our two normative cohorts using such tools, we would caution against assuming self-reported ADHD symptoms reflect ADHD, without measuring other types of disorder especially disorders of impulsivity (e.g. gambling disorder), and controlling for them.

## Disclosures and funding

Dr. Cortese declares honoraria and reimbursement for travel and accommodation expenses for lectures from the following non-profit associations: Association for Child and Adolescent Central Health (ACAMH), Canadian ADHD Alliance Resource (CADDRA), British Association of Pharmacology (BAP), and from Healthcare Convention for educational activity on ADHD. Dr. Grant has received research grants from the TLC Foundation for Body-Focused Repetitive Behaviors, the 10.13039/100001455American Foundation for Suicide Prevention, Takeda, Neurocrine, Biohaven, and 10.13039/100014934Avanir Pharmaceuticals. He receives yearly compensation for acting as editor-in-chief of the Journal of Gambling Studies and has received royalties from Oxford University Press, American Psychiatric Publishing, Inc., Norton Press, and McGraw Hill. Dr. Grant has received a research grant from the TLC Foundation for Body-Focused Repetitive Behaviors. Dr. Chamberlain consults for Promentis; and receives a stipend from Elsevier for editorial work. Dr. Chamberlain's research was funded by a 10.13039/100010269Wellcome Trust Clinical Fellowship (110049/Z/15/Z & 110049/Z/15/A).
